# HSF1 promotes endometriosis development and glycolysis by up-regulating PFKFB3 expression

**DOI:** 10.1186/s12958-021-00770-9

**Published:** 2021-06-09

**Authors:** Yixin Wang, Jing Xiu, Tingting Yang, Chune Ren, Zhenhai Yu

**Affiliations:** Department of Reproductive Medicine, Affiliated Hospital of Weifang Medical University, Weifang, Shandong Province People’s Republic of China

**Keywords:** HSF1, PFKFB3, Inhibitor, Glycolysis, Endometriosis

## Abstract

**Background:**

Endometriosis is a chronic hormonal inflammatory disease characterized by the presence of endometrial tissue outside the uterus. Endometriosis often causes infertility, which brings physical and mental pain to patients and their families.

**Methods:**

We examined the functions of heat shock factor 1 (HSF1) in endometriosis development through cell count assay, cell-scratch assay and clone formation experiments. We used quantitative real-time PCR (qRT-PCR) and Western blot (WB) to detect HSF1 expression. Glucose and lactate levels were determined using a glucose (GO) assay kit and a lactate assay kit. Furthermore, we used a HSF1 inhibitor-KRIBB11 to establish a mouse model of endometriosis.

**Results:**

Our data demonstrated that HSF1 promoted endometriosis development. Interestingly, HSF1 enhanced glycolysis via up-regulating PFKFB3 expression in endometriosis cells, which was a key glycolysis enzyme. Consistently, the HSF1 inhibitor KRIBB11 could abrogate endometriosis progression in vivo and in vitro.

**Conclusions:**

Findings indicate that HSF1 plays an important role in endometriosis development, which might become a new target for the treatment of endometriosis.

**Electronic supplementary material:**

Supplementary data are available.

**Supplementary Information:**

The online version contains supplementary material available at 10.1186/s12958-021-00770-9.

## Background

Endometriosis is a disease with features of chronic inflammation, which is defined as the functional endometrial stroma and glands outside the uterine cavity [1]. The main clinical manifestations of endometriosis are lower abdominal pain, dysmenorrhea, infertility, sexual discomfort, abnormal menstruation, local periodic pain, and bleeding. There are approximately 6–10% of women of childbearing age suffering from endometriosis in the world, and the infertility rate among them is as high as 50%, seriously affecting the women health [2]. Endometriosis is mainly affected by estrogen and progesterone, which promote endometrial tissue proliferation, survival, and inflammation [3]. In addition, the development of endometriosis is also related to progesterone resistance [4]. The most common theory of endometriosis is the implantation theory, which may be related to genetic and immune inflammatory factors [5, 6]. However, there is still no effective drug to treat endometriosis.

In eukaryotes, there are many stressors that can cause protein damage and induce an evolutionally conserved cytoprotective mechanism, the heat shock response (HSR), to maintain protein stability [7]. The heat shock factor 1 (HSF1) plays a central role in refolding or degrading intracellular proteins [8]. HSF1 is a transcription factor that can respond to endogenous and exogenous cellular stresses by inducing HSP expression, which could facilitate the refolding of misfolded proteins [9]. HSF1 also plays an important role in tumor development, which seriously affects its prognosis [10]. For example, HSF1 is highly expressed in prostate cancer, and plays its functions by increasing expression of its downstream effector HSP27 [11]. Other tumors such as colorectal cancer, breast cancer, oral cancer, and liver cancer also have a high HSF1 expression [7]. Furthermore, HSF1 can improve the tumor microenvironment to promote its survival [12]. Therefore, HSF1 can be used as a new therapeutic target. However, the roles of HSF1 in endometriosis are still largely unknown.

The increases of glucose metabolism are beneficial to the endometriosis development, and abnormal expressions of glycolysis enzymes were detected in the endometriosis cells [13]. Most normal cells mainly rely on the oxidative metabolism to produce energy, but tumor cells still choose to glycolysis pathway even in the sufficient oxygen [14]. The glycolysis pathway could produce energy quickly, which could satisfy the cell rapid proliferation [15]. Lactate produced from glycolysis promotes angiogenesis, cell invasion and immunosuppression, which promotes tumorigenesis [16]. Interestingly, endometriosis cell made a shift from oxidative phosphorylation to aerobic glycolysis, which inhibits the production of reactive oxide species and then activates survival signals [17]. Therefore, glycolysis could be considered as a target for the treatment of endometriosis. In the process of glycolysis, there is a key enzyme named 6-Phosphofructo-2-kinase/Fructose-2, 6-Biphosphatase 3 (PFKFB3), which belongs to a family of bio-functional proteins [18]. There are four members of PFKFB family, among which PFKFB3 has the highest catalytic activity in glycolysis [19]. PFKFB3, as a key enzyme of glycolysis, regulates the process of glycolysis and plays an important role in the occurrence and development of many diseases [20, 21]. Therefore, PFKFB3 has become a potential target for drug development [22]. However, the role of PFKFB3 in endometriosis remains unclear.

Endometriosis is a benign disease, but it has some clinical characteristics similar to the tumor, such as implantable, invasive, and distant metastasis. Moreover, HSF1 was previously reported to be overexpressed in endometriosis [23]. Therefore, we hypothesized that HSF1 also regulated the endometriosis development. To test this hypothesis, we manipulated HSF1 expression in endometriosis cells, and used a constructed mouse model. Our data demonstrated that HSF1 promoted endometriosis development via enhancing PFKFB3 expression. Our study provides a new idea for the clinical treatment of endometriosis by targeting HSF1.

## Materials and methods

### Cell culture and antibodies

The endometriotic epithelial cell line (11Z) was established by Professor Anna Strazinski-Powitz [24]. The human endometrial stromal cell line (ESC) was established by Dr. Krikun [25]. All cell lines were cultured in Dulbecco’s Modified Eagle Medium/Ham’s F-12 50/50 Mix (DMEM/F-12) supplemented with 10% FBS (Gibco, Carlsbad, CA, USA) with 100 μg/mL penicilin and 100 μg/mL streptomycin at 37 °C and 5% CO_2_.

Mouse anti-β-actin (A1978) was from Sigma-Aldrich, and dilution: 1:5000. Mouse anti-HSF1 (sc-17,757) was from Santa Cruz, and dilution: 1:1000. Rabbit anti-PFKFB3 (ab181861) was purchase from Abcam, and dilution: 1:2000. KRIBB11 were obtained from Med Chem Express (MCE), 50 mg/kg.

### SiRNA and transfection

The sequence of small interfering (si) RNAs against HSF1 was 5′- GCAGGUUGUUCAUAGUCAGAA-3′. The sequence of Control (Negative Control) was 5′-UUCUCCGAACGGUCACGU-3′ [26]. The transfection was performed as described previously [27].

### Western blot

The indicated cells were collected and lysed on ice using lysis buffer (Beyotime, Shanghai, China, P0013), and were centrifuged at 12000 rpm at 4 °C for 15 min. Then, 5 × loading buffer was added to the sample, and boiled for 10 min. The protein was separated by SDS-PAGE and transferred to PVDF membrane. After blocking, immunoblot assay was performed using indicated antibodies, which was performed as described previously [28].

### Quantitative real-time PCR

The isolation of total RNA from cells and the synthesis of cDNA were described above [29]. Quantitative real-time PCR used SYBR Green PCR Master Mix (Takara) with CFX96 Real-Time PCR detection system (Bio-Rad, shanghai, China).

### Cell proliferation assay

The cells were transfected with the indicated plasmids. After 24 h, the transfected cells were reseeded in 24-well plates. The cell numbers were counted every 24 h for 4 days [28].

### Colony-formation assay

The cells were transfected with indicated plasmids. After 24 h, 500 transfected cells were reseeded in new six-well plates. After cultured for 10–14 days, the cells were fixed with 4% paraformaldehyde for 15 min. Then, the cells were stained with crystal violet for 20 min, and photographed [30].

### Cell-scratch assay

The cells were transfected with indicated plasmids. After 24 h, the transfected cells were reseeded in new 6-well plates. The pipette tip was used to draw a line and washed with PBS. After 24 h, cells were photographed [31].

### Glucose consumption and lactate production

The cells were transfected with indicated plasmids. After 24 h, the transfected cells were reseeded in new 6-well plates. After 1 day, and the culture mediums were collected to determine the concentration of glucose and lactic acid using glucose (GO) assay kit (Sigma, #GAGO20-1KT) and lactate assay kit (Biovision, #K627–100). The methods were performed as described previously [28, 30].

### Animal experiments

Animal experiments have been approved by ethics Committee of Weifang Medical University. We used 5-week-old BALB/c female mice, and the donor mice (*n* = 5) were injected with estradiol benzoate to promote endometrial development. Estradiol benzoate was diluted with oil and injected intramuscularly into the thigh of donor mice, 3 μg/mouse, 2 times for 1 week. After 1 week, the uterus from donor mice was cut into pieces and intraperitoneally injected into the recipient mice. After 1 week, the mice in the experimental group (*n* = 7) were intraperitoneally injected HSF1 inhibitor KRIBB11, and the mice in the control group (n = 7) were injected with normal saline, 2 times a week for 1 month. Then, the mice were sacrificed to observe the endometrial lesion.

### Tissue collection and immunohistochemistry

All tissues were obtained from endometriosis mouse model. The sections were embedded in paraffin, dried and dewaxed with xylene, then hydrated in ethanol. Antigen extract was heated in a microwave oven for 30 min, and incubated with 3% H_2_O_2_ for 20 min to block endogenous peroxidase. Primary antibody was incubated overnight at 4 °C, and secondary antibody was incubated in the next day. After staining with DAB, the nucleus was stained with hematoxylin. Then, it is dehydrated in ethanol and xylene. The immunohistochemistry was performed as described previously [27]. The immunostaining intensity was quantified using the Image J [32].

### Statistical analysis

All statistical analyses were done using Graphpad Prism 5.0 software. The statistical analyses were presented as mean ± SEM, and performed by two-tailed unpaired Student’s t-test. *P* values < 0.05 were considered to be statistically significant. n.s. was not significant.

## Results

### HSF1 promotes cell proliferation, cell migration and clone formation in endometriosis cells

**Fig. 1:**
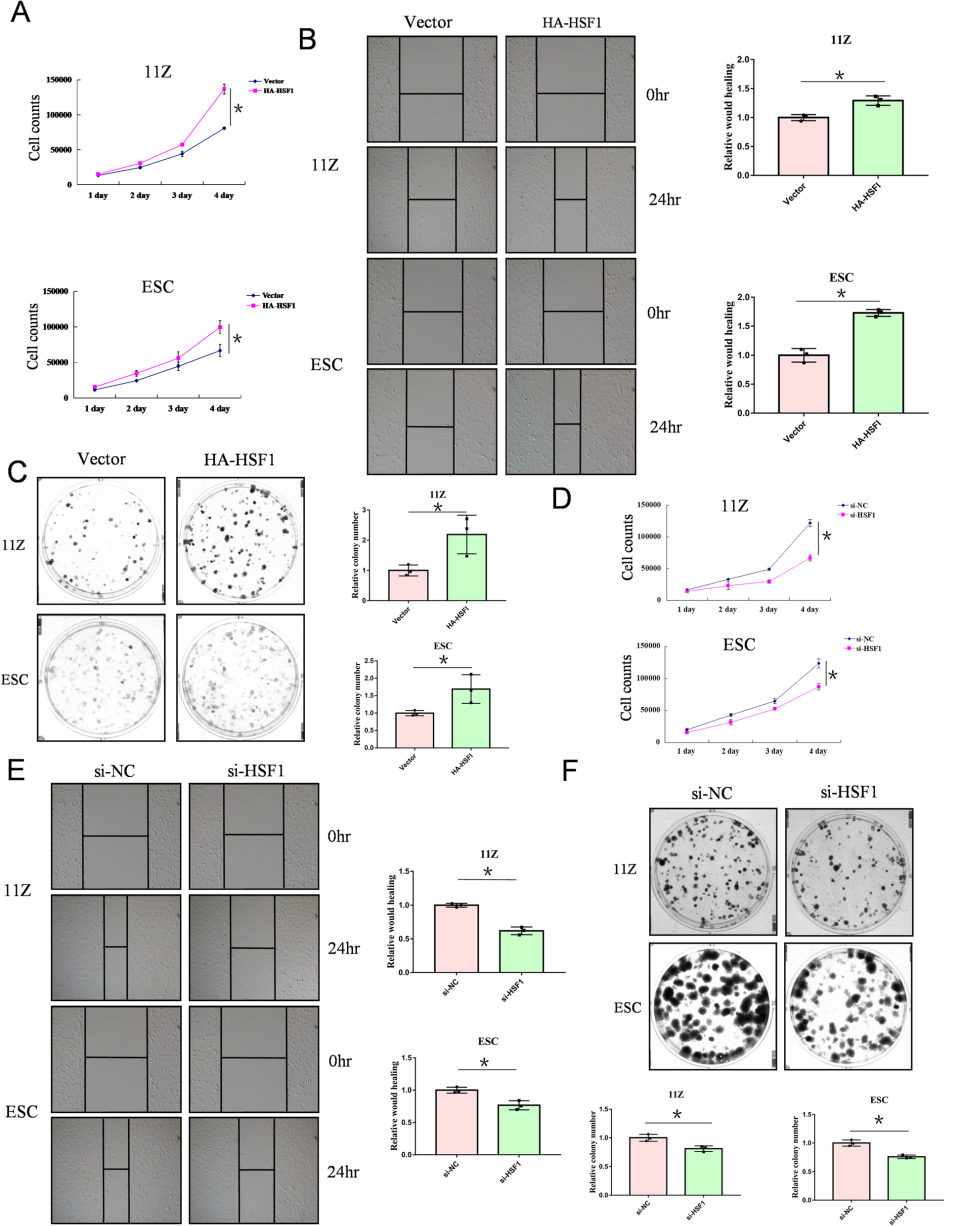
HSF1 promotes the cell proliferation, cell migration and clone formation in endometriosis cells. (**A**) 11Z and ESC cells were transfected with HA-tagged HSF1 or empoty vector. After one day, cells were re-plated in 24-well plates, and cell counts were performed every 24 h to analyses cell growth. (**B**) 11Z and ESC cells were transfected with HA-tagged HSF1 or empoty vector. After one day, cells were re-plated in 6-well plates to perform scratch test assay. (**C**) 11Z and ESC cells were transfected with HA-tagged HSF1 or empoty vector. After one day, cells were re-plated in 6-well plates, and were cultured for 10–14 days to observe the cell clone formation. (**D**-**F**) 11Z and ESC cells were transfected with siRNA-HSF1 or NC. Cell counting, scratching, and cloning were performed. All date are mean ± SD of three independent experiments (*P < 0.05)

To determine whether HSF1 plays an important role in endometriosis, we manipulated HSF1 expression in endometriosis cells. We found that HSF1 overexpression significantly promoted cell proliferation in endometriosis cells (Fig. 1A). Moreover, cell-scratch tests and clone formation experiments revealed that HSF1 overexpression promoted cell migration and growth in endometriosis cells (Fig. 1B and C). Conversely, HSF1 knockdown inhibited the growth of endometriosis cells (Fig. 1D and F), and inhibited cell migration (Fig. 1E). These findings suggest that HSF1 positively regulates cell proliferation and migration in endometriosis cells.

### HSF1 enhances glycolysis in endometriosis cells

**Fig. 2:**
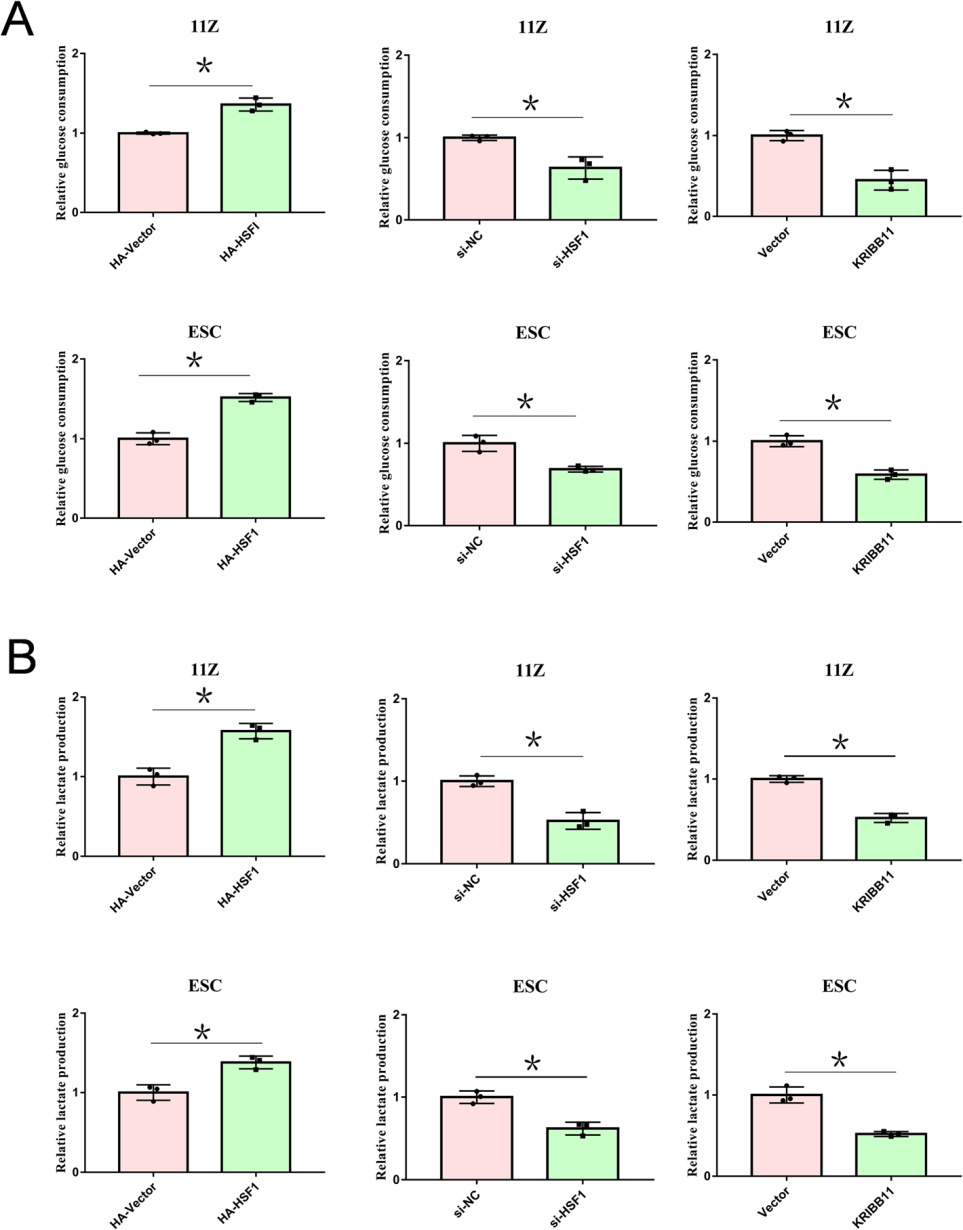
HSF1 enhances glycolysis in endometriosis cells. (**A**, **B**) 11Z and ESC cells were transfected with HA-tagged HSF1 or empoty vector, siRNA or NC. Cells were re-plated in 6-well plates. After 24 h, glucose and lactic acid concentrations in culture medium were determine using glucose and lactic acid kits. 11Z and ESC cells were cultured with KRIBB11 for 24 h, and the concentration of glucose and lactic acid in the super-medium was determined. All date are mean ± SD of three independent experiments (*P < 0.05)

Endometriosis cells need high glycolysis in the process of rapid metastasis and growth [13]. To determine the functions of HSF1 in glycolysis, we overexpressed or knocked down HSF1 in endometriosis cells. Interestingly, we found that HSF1 could increase both glucose consumption and lactate production (Fig. 2A and B). Subsequently, to determine whether HSF1 inhibitor KRIBB11 could suppress glucose metabolism, we cultured endometriosis cells with KRIBB11. Consistently, KRIBB11 reduced the glucose consumption and lactic acid generation in endometriosis cells (Fig. 2A and B). These data show that HSF1 enhances glycolysis in endometriosis cells.

### HSF1 promotes PFKFB3 expression in endometriosis cells

**Fig. 3:**
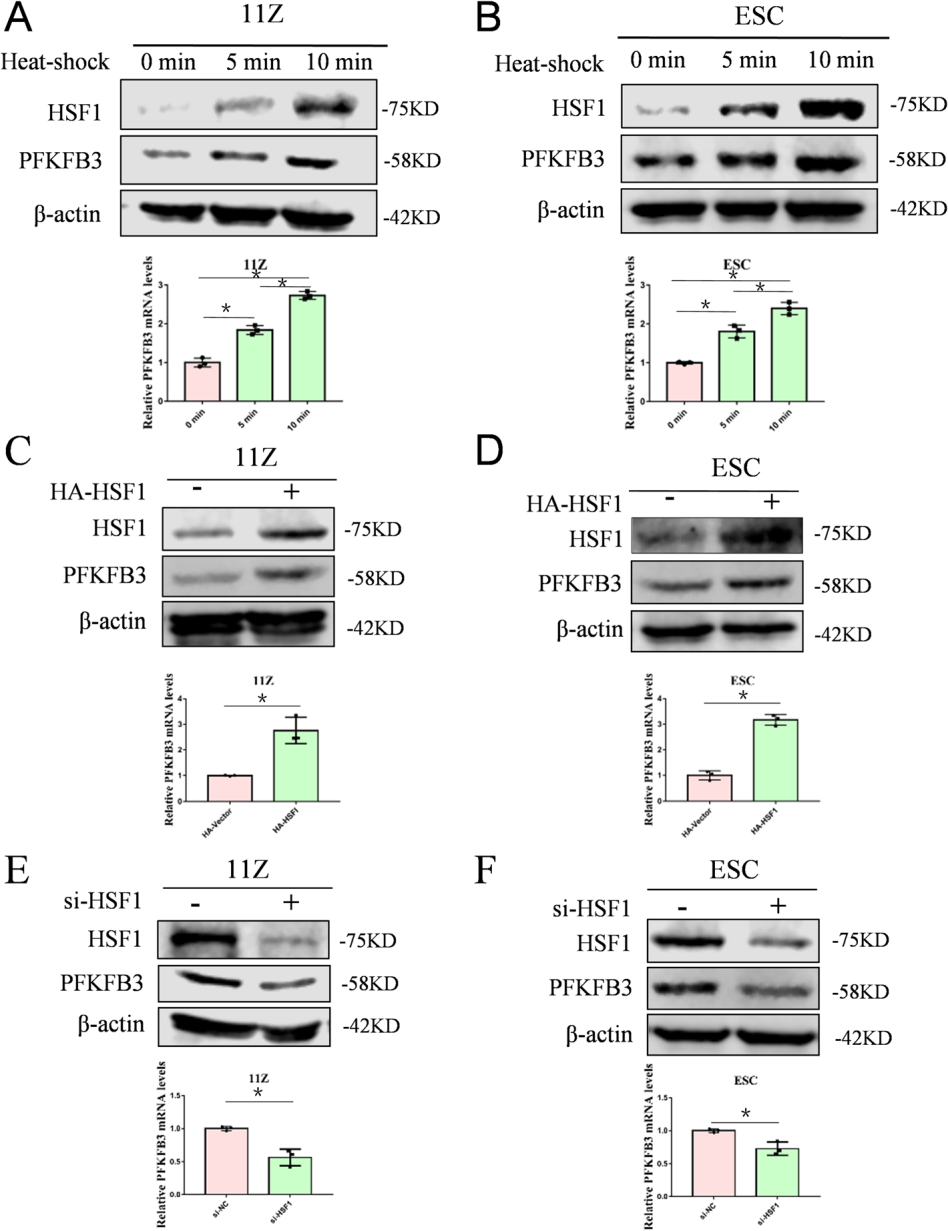
HSF1 promotes PFKFB3 expression in endometriosis cells. (**A**, **B**) 11Z and ESC cells were heat shocked in a time-dependent manner. The expression of PFKFB3 was determined by Western blot and qRT-PCR. (**C**, **D**) 11Z and ESC cells were transfected with HA-tagged HSF1 or empoty vector, and the expressions of PFKFB3 were detected. (**E**, **F**) 11Z and ESC cells were transfected with siRNA-HSF1 or NC. The expressions of PFKFB3 were detected after 2 days. All date are mean ± SD of three independent experiments (*P < 0.05)

In the previous experiment of the current study, we showed that HSF1 promotes glycolysis in endometriosis cell. Therefore, we hypothesized that regulation of glycolysis by HSF1 might depend on key glycolytic enzymes. So we selected three key enzymes in glycolysis to verify our hypothesis, PFKFB3, PKM2 and HK2. Exposing cells to heat-shock in a time-dependent manner, the PFKFB3 expression were increased (Fig. 3A and B). But heat-shock activation had little effect on the *PKM2* and *HK2* expressions (Supplementary Fig. 1A and B). In addition, overexpression HSF1 increased PFKFB3 expression (Fig. 3C and D). Conversely, HSF1 knockdown resulted in a decrease in PFKFB3 expression (Fig. 3E and F). Taken together, our results indicate that HSF1 promotes PFKFB3 expression in endometriosis cells.

### KRIBB11 inhibits endometriosis cell growth by targeting HSF1

**Fig. 4:**
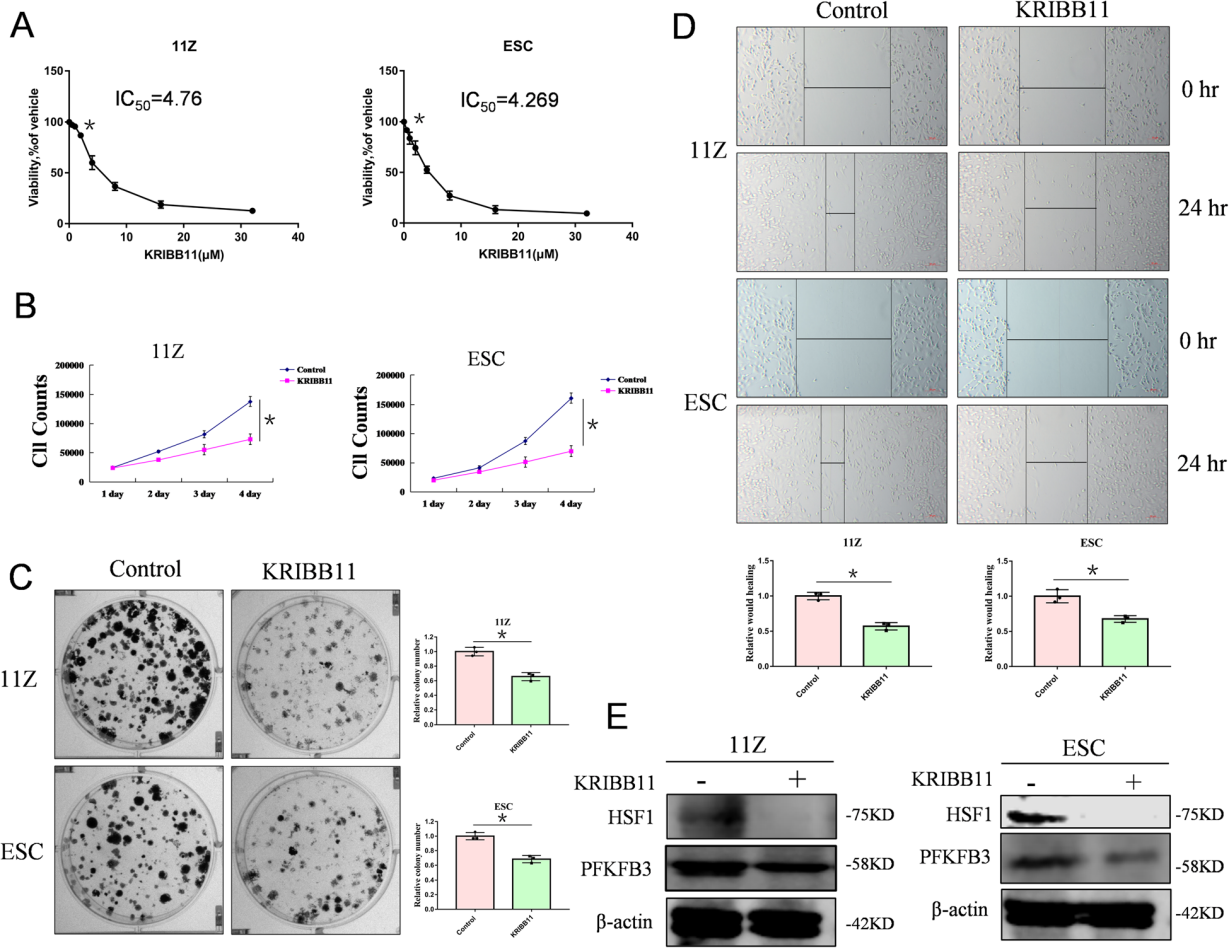
KRIBB11 inhibits endometriosis cell growth by targeting HSF1. (**A**) 11Z and ESC cells were plated in 24-well plates. KRIBB11 was given in a concentration-dependent manner, and the IC_50_ value of the drug was measured by cell counting. (**B**) 11Z and ESC cells were plated in 24-well plates, and the experimental group treated with HSF1 inhibitor KRIBB11. The cell counts were performed every 24 h for 4 days. (**C**) 11Z and ESC cells were plated in 6-well plates, and the experimental group treated with HSF1 inhibitor KRIBB11. After 10–14 days, the cell clones were analyzed. (**D**) 11Z and ESC cells were plated in 6-well plates, and the scratches were made by pipette tip. Experimental group was treated with HSF1 inhibitor KRIBB11. After 24 h, the wound healing was analyzed. (**E**) 11Z and ESC cells were plated in 6-well plates. The experimental group was treated with HSF1 inhibitor KRIBB11. After 24 h, the cells are lysed for westen blot. All date are mean ± SD of three independent experiments (*P < 0.05)

KRIBB11, a specific inhibitor of HSF1, effectively inhibits HSF1 activity, leading to cell cycle arrest in the G2/M phase, cell apoptosis, and inhibition of tumor cell proliferation [33]. Cells were seeded in 24-well plates and given an increasing concentration of KRIBB11. The IC_50_ values of two cell lines were measured (Fig. 4A). As we expected, KRIBB11 inhibited the growth of endometriosis cells (Fig. 4B and C). Cell-scratch tests indicated that KRIBB11 inhibited the migration of endometrial cells (Fig. 4D). Western blot showed that PFKFB3 protein level was reduced after HSF1 inhibition by KRIBB11 (Fig. 4E). Thus, these data reveal that HSF1-specific inhibitor KRIBB11 reduces the key glycolytic enzyme PFKFB3 expression by inhibiting HSF1, and ultimately inhibits endometriosis cell growth.

### KRIBB11 plays a therapeutic role in a mouse model of endometriosis

**Fig. 5:**
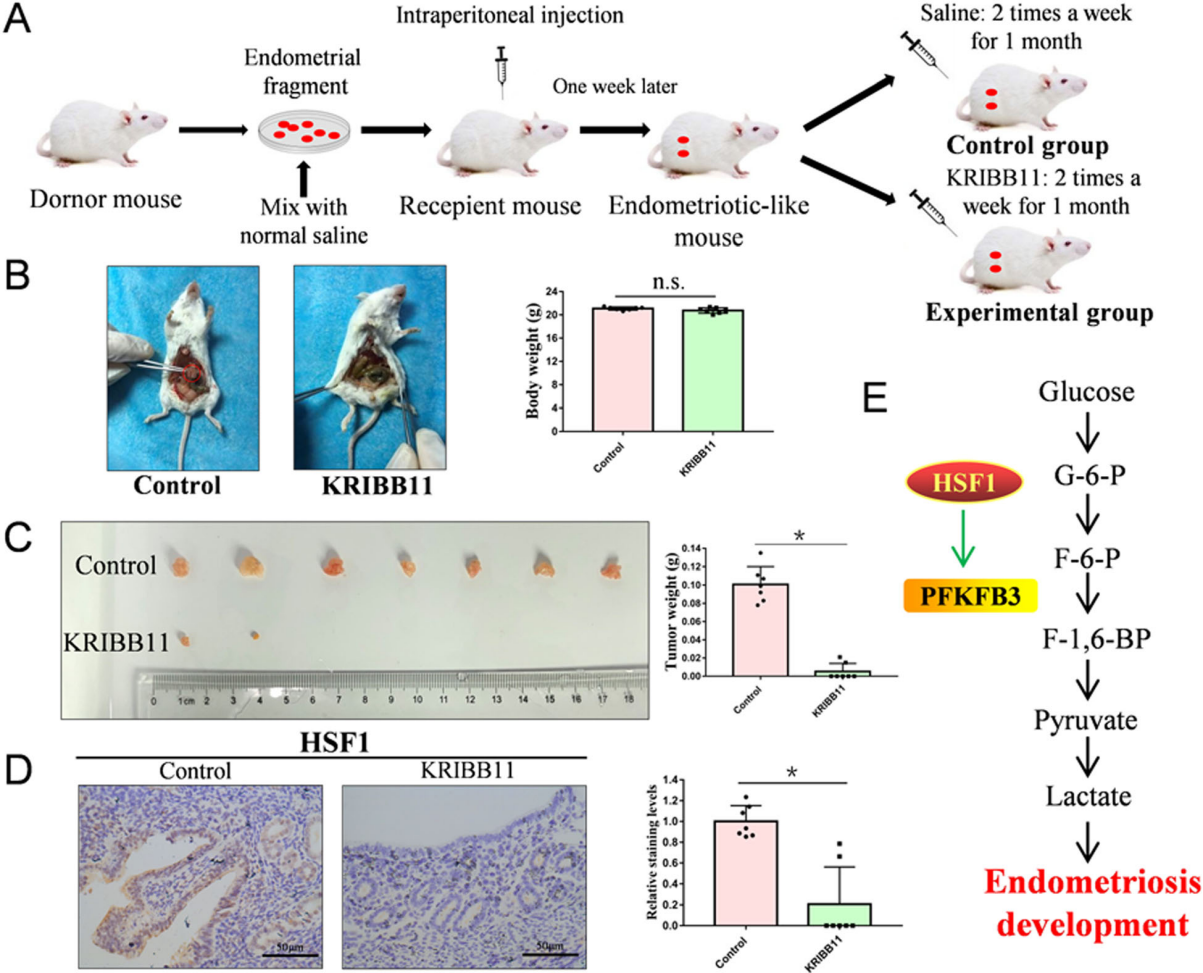
KRIBB11 plays a therapeutic role in a mouse model of endometriosis. (**A**) Endometriosis model was established using 5-week-old BALB/c female mice. (**B**) Mice were sacrificed, the endometriosis tissues and the weight of mice were analyzed (*P < 0.05). (**C**) The size of the ectopic tissues was observed and heterotopic tissues were weighed (*P < 0.05). (**D**) Immunohistochemical staining was performed to determine the HSF1 expression in the control group and the experimental group (Scale bars, 50 μm). Quantitative analyses of HSF1 expression was performed. (**E**) HSF1 promoted glycolysis by up-regulating the expression of PFKFB3, which induced the development of endometriosis

To determine whether KRIBB11 regulates endometriosis cell growth in vivo, the endometria of donor mice were cut up and intraperitoneally injected into recipient mice. After one week, a mouse model of endometriosis was established, the experimental group was treated with KRIBB11 and the control group was injected with normal saline (Fig. 5A). At the end, the mice were sacrificed to observe ectopic lesion. Interestingly, all control mice were observed the endometriosis tissues, but only two in the mice treated with KRIBB11 were observed (Fig. 5B). Ectopic lesions from mice treated with KRIBB11 grew significantly slower than those in control group. Consistently, the weight of ectopic lesions from mice treated with KRIBB11 was lower than in the control group (Fig. 5C). Using immunohistochemical staining, we found that HSF1 expression was significantly lower in the mice treated with KRIBB11 (Fig. 5D). Taken together, HSF1-specific inhibitor KRIBB11 plays a therapeutic role in the mouse model of endometriosis.

## Discussion

Endometriosis is an age-related disease of the reproductive system, and its prevalence is up to 10% in premenopausal women worldwide [6]. The diagnosis of endometriosis is difficult, because experienced obstetricians and gynecologists are required to correctly assess the clinical symptoms of this disease [34]. In recent years, more studies have been published on how to treat endometriosis. However, the treatment of endometriosis is still a clinical challenge, which causes increased burdens to women of childbearing age. Because endometriosis cells have similar characteristics of invasion and metastasis with tumor cells, and HSF1 is a carcinogen promoting tumor progression, so we speculate that HSF1 plays a similar role in the occurrence and development of endometriosis. Our hypothesis is supported by a series of experiments. Our data show that HSF1 promotes endometriosis development, and enhances glycolysis via up-regulating PFKFB3 in endometriosis cells. In mice, we treat with HSF1 inhibitor KRIBB11, which could effectively inhibit endometriosis development. Taken together, HSF1 is a promising target for endometriosis.

HSF1 is discovered in 1984 as the main regulator of HSR. HSF1 is activated after cell stress, which leads to the HSP expression to protect cells. After heat shock, HSF1 is phosphorylated, trimerized, and transferred to the nucleus to induce chaperone gene expression by binding to DNA sequence motifs known as heat shock elements (HSEs) [35]. HSF1 transcriptional activity mainly depends on the formation of trimer in the nucleus, and post-translational modifications also could regulate its transcriptional activity, such as acetylation, phosphorylation, and methylation [36]. Specially, HSF1 is found to play an important role in multiple cancers, which promotes cell invasion, migration, and proliferation of tumor cells [37]. Cancer cells rely on HSR to support their rapid growth and counteract the harmful mutations [38]. Previous studies have demonstrated that HSF1 is highly expressed in endometrial carcinoma and is closely related to endometrial invasion, which leads to a poor prognosis in estrogen receptor-positive tumors [39]. Endometriosis is also a highly estrogen-dependent disease [40]. Our study demonstrates that HSF1 plays a crucial role in endometriosis development, which is consistent with previous studies.

Metabonomics can be used as a diagnostic tool to study the metabolic changes under the physiological or pathological state of disease [41]. The lipid metabolism [42], amino acid metabolism [43], and glucose metabolism [13] in patients with endometriosis are increased. Women with endometriosis have high levels of cholesterol compared to a control group without endometriosis [42]. Quantitative analysis of lipid metabolites shows that the concentrations of phosphatidylcholine and phosphatidylserine in patients with early endometriosis (I-II) are decreased, while the concentrations of phosphatidylic acid are increased [44]. Endometriosis is largely determined by estrogen synthesis and metabolism genetic factors, which increase the risk of developing endometriosis [45]. Similar to tumor cells, Warburg Effect also occurs in stromal cells of endometrial tissues, which increases glucose consumption and lactate production [13]. In addition, increased glucose metabolism may be the cause of excessive reactive oxygen species in endometriosis [46]. Moreover, the expression of both aerobic and anaerobic glycolytic markers was increased in endometriosis patients, which ultimately contribute to endometriosis development [47]. As a rate-limiting enzyme in glycolysis, PFKFB3 has the highest kinase activity to guide glucose into glycolysis. In our study, we find PFKFB3 is highly expressed and promotes glycolysis in endometriosis cells, which is consistent with previous study that glycolysis promotes endometriosis development. Taken together, our findings provide some new insights into the functions of HSF1/PFKFB3 axis in endometriosis development, which is as a new target to treat endometriosis (Fig. 5E).

## Conclusions

Our data show that HSF1 plays an important role in the development of endometriosis. HSF1 can regulate glycolysis process by up-regulating the expression of PFKFB3 and ultimately promote the growth of endometriosis, while HSF1 specific inhibitors can inhibit the above effects. This will provide a new way of thinking for the treatment of endometriosis in the future.

## Supplementary Information

**Additional file 1: Supplementary Figure 1.** Heat-shock activation had little effect on the *PKM2* and *HK2* expressions. (A) 11Z cells were heat-shocked for 0 or 10 min, and qRT-PCR was performed to analyze the *HK2* and *PKM2* mRNA levels. (B) ESC cells were heat-shocked for 0 or 10 min, and qRT-PCR was performed to analyze the *HK2* and *PKM2* mRNA levels.

## Data Availability

The data used in this study are available from the corresponding author on reasonable request.

## Abbreviations

HSF1: Heat shock factor 1
HSR: Heat shock response
HSP: Heat shock protein
HSEs: heat shock elements
PCR: Polymerase chain reaction
qRT-PCR: Quantitative real-time PCR
WB: Western blot
PFKFB3: 6-Phosphofructo-2-kinase/Fructose-2, 6-Biphosphatase 3
DMEM/F-12: Dulbecco’s Modified Eagle Medium/Ham’s F-12 50/50 Mix
FBS: Fetal bovine serum
CO_2_: Carbon dioxide
RNA: Ribonucleic acid
siRNA: Small interfering RNA
DNA: Deoxyribonucleic acid
cDNA: Complementary DNA
PBS: Phosphate buffer saline
PKM2: pyruvate kinase 2
HK2: Hexokinase 2
IC_50_: 50% inhibiting concentration
p-TEFb: Positive transcription elongation factor b
SEM: Standard error of the means
SD: Standard deviation
n.s.: Not significant
