# Supplementary material for: HSF1 promotes endometriosis development and glycolysis by up-regulating PFKFB3 expression
Source: Reprod Biol Endocrinol. 2021 Jun 9;19:86. doi: 10.1186/s12958-021-00770-9 (PMC8188696; doi:10.1186/s12958-021-00770-9)
Supplement: Supplementary file 1 — Additional file 1: Supplementary Figure 1. Heat-shock activation had little effect on the PKM2 and HK2 expressions. (A) 11Z cells were heat-shocked for 0 or 10 min, and qRT-PCR was performed to analyze the HK2 and PKM2 mRNA levels. (B) ESC cells were heat-shocked for 0 or 10 min, and qRT-PCR was performed to analyze the HK2 and PKM2 mRNA levels. [file 12958_2021_770_MOESM1_ESM.doc]

**Supplementary Data**


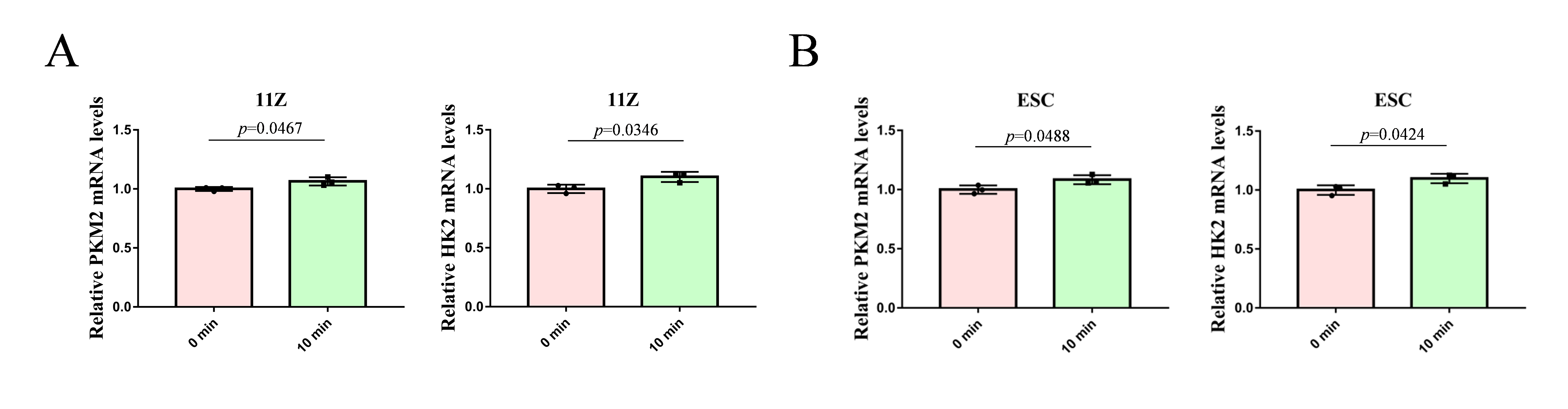


**Supplementary Figure 1. (A)** 11Z cells were heat-shocked for 0 or 10min, and qRT-PCR was performed to analyze the *HK2* and *PKM2* mRNA levels. **(B)** ESC cells were heat-shocked for 0 or 10min, and qRT-PCR was performed to analyze the *HK2* and *PKM2* mRNA levels.
